# Treatment patterns and a prognostic scoring system for elderly acute myeloid leukemia patients: a retrospective multicenter cohort study in China

**DOI:** 10.20892/j.issn.2095-3941.2020.0474

**Published:** 2021-08-27

**Authors:** Chunli Zhang, Wei Wan, Shuai Zhang, Jingwen Wang, Ru Feng, Jiangtao Li, Junyue Chai, Hebing Zhou, Liru Wang, Yuping Zhong, Xiaodong Mo, Mengzhu Shen, Hongmei Jing, Hui Liu

**Affiliations:** 1 Department of Hematology, Beijing Hospital, National Center of Gerontology, Institute of Geriatric Medicine, Chinese Academy of Medical Sciences, Beijing 100730, China; 2 Department of Hematology, Peking University Third Hospital, Beijing 100191, China; 3 Department of Hematology, Beijing Tongren Hospital, Capital Medical University, Beijing 100730, China; 4 Department of Hematology, Beijing No. 6 Hospital, Beijing 100007, China; 5 Department of Hematology, Beijing Luhe Hospital, Capital Medical University, Beijing 101100, China; 6 Department of Hematology, Fuxing Hospital, Capital Medical University, Beijing 100038, China; 7 Department of Hematology, Beijing Chao-Yang Hospital, Capital Medical University, Beijing 100043, China; 8 Peking University People’s Hospital, Peking University Institute of Hematology, National Clinical Research Center for Hematologic Disease, Beijing Key Laboratory of Hematopoietic Stem Cell Transplantation, Beijing 100044, China

**Keywords:** Acute myeloid leukemia, chemotherapy, comorbidity, elderly, geriatric assessment

## Abstract

**Objective:**

Acute myeloid leukemia (AML) is primarily a malignant disorder affecting the elderly. We aimed to compare the outcomes of different treatment patterns in elderly AML patients and to propose a prognostic scoring system that could predict survival and aid therapeutic decisions.

**Methods:**

Patients aged ≥ 60 years who had been diagnosed with AML at 7 hospitals in China were enrolled (*n* = 228). Treatment patterns included standard chemotherapy, low intensity therapy, and best supportive care (BSC).

**Results:**

The early mortality rates were 31%, 6.8%, and 6.3% for the BSC, low intensity therapy, and standard chemotherapy groups, respectively. The complete remission rate of the standard chemotherapy group was higher than that of the low intensity therapy group. The median overall survival (OS) was 561 days and 222 days for the standard chemotherapy and low intensity therapy groups, respectively, and were both longer than that of the BSC group (86 days). Based on multivariate analyses, we defined a prognostic scoring system that enabled classification of patients into 3 risk groups, in an attempt to predict the OS of patients receiving chemotherapies and low intensity therapies. Low and intermediate risk patients benefited more from standard chemotherapies than from low intensity therapies. However, the median OS was comparable between standard chemotherapies and low intensity therapies in high risk patients.

**Conclusions:**

Our prognostic scoring system could predict survival and help select appropriate therapies for elderly AML patients. Standard chemotherapy is important for elderly AML patients, particularly for those categorized into low and intermediate risk groups.

## Introduction

Acute myeloid leukemia (AML) is the most common type of acute leukemia in adults. The incidence of AML increases with age, with a median age at diagnosis of 68–72 years^1–3^. Thus, AML is primarily a malignant disorder of the elderly. A diagnosis of AML predicts the shortest median overall survival (OS: 2.67 months) and the lowest estimated 1 year OS (21.8%) by cancer type when diagnosed at ages ≥ 65 years^3^. Thus, elderly AML patients should be carefully managed.

Chemotherapy is the fundamental therapy for AML, and complete remission (CR) and long-term survival mostly requires standard chemotherapy^4–6^. However, considering that the benefits of chemotherapy might be compromised by chemotherapeutic toxicities, the proportion of patients intended to receive intensive chemotherapies has decreased with age, with more than 50% of the elderly AML patients not receiving chemotherapies^5,6^. In addition, the median OS of the elderly AML patients receiving chemotherapy was only 6 months in a population-based study (*n* = 5,480)^7^. However, to the best of our knowledge, no study has compared the efficacies of standard chemotherapy and best supportive care (BSC) in elderly patients with AML in China, so knowledge about the benefits of standard chemotherapies compared to BSC for Chinese patients is still unclear.

In addition, identifying patients who would be suitable to receive standard chemotherapy was another critical factor to improve the survival of elderly AML patients^8^. Geriatricians developed geriatric assessment (GA) methods for elderly patients with cancers^9,10^, and several studies have shown that GA could predict the clinical outcomes of AML patients^11–17^. However, there is no single gold standard measure of GA in AML patients. In addition, GA does not include the disease characteristics (e.g., hyperleukocytosis) in elderly AML patients. Thus, there is no well-accepted, comprehensive model that considers both patient and disease characteristics in therapeutic decisions for elderly AML patients. In addition, emerging new drugs have played an increasingly important role in the treatment of AML, providing another therapeutic option for elderly AML patients^18^. The toxicities of these drugs are relatively less, and most of them are well-tolerated (i.e., low intensity therapy). A large data analysis of treatment patterns and outcomes among elderly AML patients showed that the median OS of low intensity therapy was better than that of BSC but poorer than that of standard chemotherapy in the United States^6^. However, the role of low intensity therapy in elderly AML patients has not been definitely identified in China.

In this multicenter study, we therefore aimed to compare the efficacies of different treatment patterns in elderly AML patients. We also proposed a prognostic scoring system that could predict survival and help therapeutic decisions for these patients.

## Materials and methods

### Study population

This was a retrospective multicenter cohort study of elderly patients with AML (non-acute promyelocytic leukemia) at 7 hospitals in Beijing, China (Beijing Hospital, Peking University Third Hospital, Beijing Tongren Hospital, Beijing No. 6 Hospital, Beijing Luhe Hospital, Fuxing Hospital, and Beijing Chao-Yang Hospital). We included patients with ages ≥ 60 years who had been diagnosed with AML between April 1, 2010 and June 30, 2019. Patients without complete medical information were excluded. The final follow-up visits for survival analyses were conducted on March 31, 2020. The study was performed in accordance with the Declaration of Helsinki, and the protocol was approved by the Local Ethics Committees of each participating hospital (Approval No. 2018BJYYEC-154-02). Sixty patients had been previously reported^19^ and all of them were followed-up in the present study.

### Data collection

We retrospectively reviewed the medical records that had been collected at the time of diagnosis of leukemia, including age, gender, diagnosis, cytogenetics, white blood cell, hemoglobin, platelet, serum albumin, serum creatinine, Eastern Cooperative Oncology Group (ECOG) performance status (range: 0–5), instrumental activities of daily living (IADL) scales (range: 0–8, higher score indicated less need for assistance)^20^, activity of daily living scales (ADL; range: 0–6; a higher score indicated less need for assistance)^21^, Charlson Comorbidity Index (CCI)^22^, and Mini-mental State Examination (MMSE; using 26 as the cut-off point)^23,24^. Only patients who had all of the above medical information were included in the study (**Supplementary Figure S1**).

As previously reported, Liu et al.^19,25^ proposed the IACA index, which could predict the clinical outcomes of elderly patients with diffuse large B-cell lymphoma (DLBCL) and AML. The IACA index included the following variables: IADL scales (8 *vs.* 6–7 *vs.* ≤ 5), age (> 75 years *vs.* ≤ 75 years), comorbidities (CCI score of ≥ 3 *vs*. < 3), and hypoalbuminemia (< 3.4 g/dL *vs*. ≥ 3.4 g/dL). Patients were categorized into low (0 points), intermediate (1–2 points), and high risk (≥ 3 points) groups (**Supplementary Table S1**)^19^.

### Treatments

Standard chemotherapy protocols for induction therapy always consists of an anthracycline plus cytosine arabinoside (“3 plus 7”, or similar), according to Chinese guidelines and estimated patient status^26^. However, the choice of chemotherapy protocols should also be based on each center’s competence and experience. Thus, the chemotherapy protocols varied by center in the present study. Patients could also receive low intensity therapies, which included hypomethylating agent (HMAs)-based therapies or targeted drugs. If the patients did not want to receive standard induction chemotherapies or low intensity therapies, they were given BSC to maximize their quality of life. Hydroxycarbamide or low dose cytarabine to reduce the leukocyte count in patients with hyperleukocytosis was also considered as BSC. Patients were required to receive a medical evaluation at each center or the local hospital at least once a month.

### Definitions and assessments

Toxicities of therapies were rated according to the World Health Organization criteria^27^. High leukocyte counts at diagnosis were defined as ≥ 50 × 10^9^ cells/L. The cytogenetic classification was based on the National Comprehensive Cancer Network criteria^28^. Response evaluation was in accordance with the common international criteria^29^. Early mortality was defined as death within 8 weeks after AML initial diagnosis^5,7^. OS was defined as the time from the date of diagnosis to the date of death from any cause or last follow-up for censored patients. Patients without OS events were censored based on the last date with valid information for the end points.

### Statistical analysis

Continuous variables were compared using the Mann-Whitney *U* test; while categorical variables were compared using χ^2^ and Fisher’s exact tests. The probabilities of OS were calculated using the Kaplan-Meier estimator. Hazard ratios (HRs) for OS were estimated using Cox proportional hazards regression with a backward stepwise model selection approach. Factors included in the regression model were gender (female *vs.* male), hyperleukocytosis at diagnosis (≥ 50 × 10^9^ cells/L *vs.* < 50 × 10^9^ cells/L)^30^, secondary AML (yes *vs.* no), cytogenetics (poor *vs.* intermediate *vs.* favorable), ECOG performance status (≥ 2 *vs*. < 2), ADL (≤ 4 *vs*. 6 *vs*. 5), MMSE (≤ 26 *vs*. > 26), IACA index (high risk *vs.* intermediate risk *vs.* low risk), and therapy patterns (standard chemotherapy *vs*. low intensity therapy *vs*. BSC). All reported *P*-values were based on two-sided tests, and significance was set at *P* = 0.05. Data analyses were primarily conducted using SPSS statistical software for Windows (SPSS, Chicago, IL, USA) and the R-software package, version 3.6.2; (http://www.r-project.org).

## Results

### Patient characteristics

We enrolled 228 patients (**Table 1**; **Supplementary Figure S1**). Among the patients receiving low intensity therapy, most of them received decitabine-based therapies (*n* = 42) while the others received sorafenib (*n* = 1) or venetoclax (*n* = 1) as monotherapies. The characteristics of comorbidities are shown in **Supplementary Table S2**.

**Table 1:** Patient characteristics

Characteristics	Total (*n* = 228)
Age at diagnosis (years)	71 (60–91%)
Gender	
Male	143 (62.7%)
Female	85 (37.3%)
ADL score	
6	168 (73.7%)
5	26 (11.4%)
≤ 4	34 (14.9%)
IADL score	
8	119 (52.2%)
6–7	58 (25.4%)
≤ 5	51 (22.4%)
ECOG score, ≥ 2	83 (36.4%)
MMSE score, ≤ 26	73 (32.0%)
Charlson Comorbidity Index, ≥ 3	36 (15.8%)
WBC at diagnosis (10^9^ cells/L)	7.5 (0.3–460.3)
WBC at diagnosis, ≥ 50 × 10^9^ cells/L	51 (22.4%)
Hemoglobin at diagnosis, (g/L)	80 (36–159)
Platelet at diagnosis, (10^9^ cells/L)	52 (2–1,559)
FAB Subtype	
M0	4 (1.8%)
M1	10 (4.4%)
M2	138 (60.5%)
M4	44 (19.3%)
M5	26 (11.4%)
M6	6 (2.6%)
Cytogenetic at diagnosis	
Favorable	14 (6.1%)
Intermediate	178 (78.1%)
Poor	36 (15.8%)
Secondary AML	48 (21.1%)
Serum albumin at diagnosis (g/dL)	3.7 (2.2–8.8)
Serum albumin < 3.4 (g/dL)	65 (28.5%)
Serum creatinine at diagnosis (μmol/L)	79 (33–567)
IACA index^†^	
Low risk (score 0)	61 (26.8%)
Intermediate risk (score 1 to 2)	114 (50.0%)
High risk (score ≥ 3)	53 (23.2%)
Therapies	
Best supportive care	42 (18.4%)
Low-intensity therapy^‡^	44 (19.3%)
Standard chemotherapy	142 (62.3%)
Duration of follow-up (days)	265 (3–4,075)

Data was present as *n* (%) or median (range). ADL, activities of daily living; AML, acute myeloid leukemia; ECOG, Eastern Cooperative Oncology Group; IADL, instrumental activity of daily living; IACA index, IADL scales, Age, Comorbidities, and Albumin index; MMSE, Mini-mental State Examination; WBC, white blood cell. ^†^IACA index included the following variables: age (> 75 years *vs*. ≤ 75 years), IADL scale (8 *vs.* 6 to 7 *vs.* ≤ 5), hypoalbuminemia (< 3.4 g/dL *vs*. ≥ 3.4 g/dL), and comorbidities (CCI score of ≥ 3 *vs*. < 3). Patients were categorized into low (0 point), intermediate (1–2 points), and high risk (≥ 3 points) groups. ^‡^Low intensity therapy included decitabine-based therapies (*n* = 42), sorafenib alone (*n* = 1), and venetoclax alone (*n* = 1).

### Early mortality, toxicities, and CR

**Figure 1:**
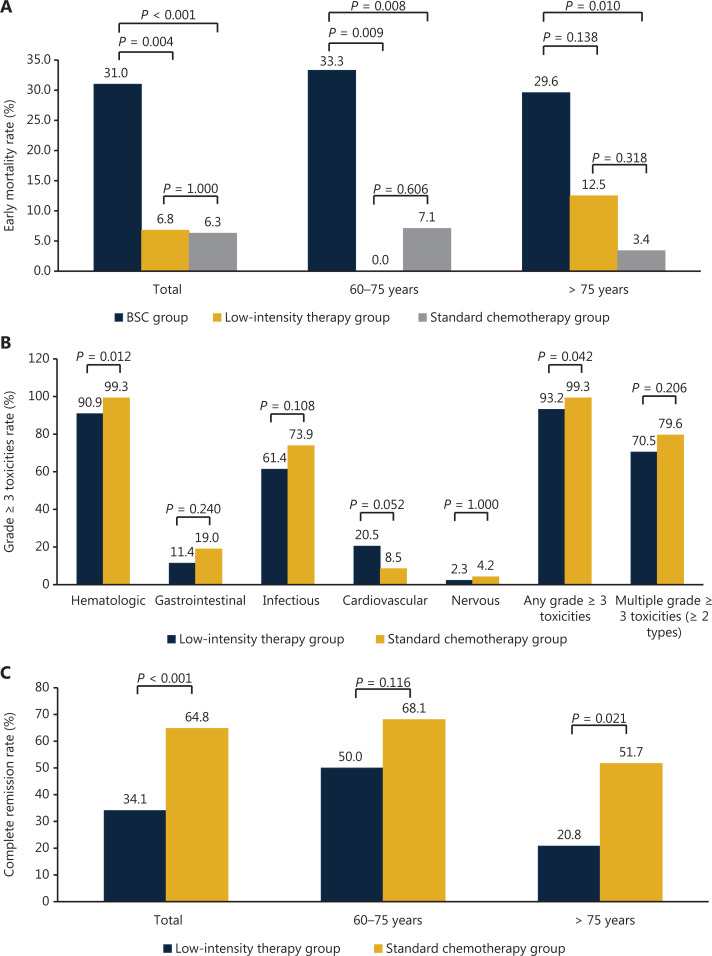
Early mortality, therapeutic toxicities, and complete remission after different treatment patterns of (A) early mortality (B) therapeutic toxicities, and (C) complete remission.

Patients who received BSC had the highest rate of early mortality (31%). However, the early mortality rates were comparable between the standard chemotherapy (6.3%) and low intensity therapy groups (6.8%). A similar situation was observed in patients aged 60–75 years and > 75 years (**Figure 1A**).

Patients who received standard induction chemotherapies showed higher rates of grade ≥ 3 hematological toxicities (99.3% *vs*. 90.9%; *P* = 0.012) and any grade ≥ 3 toxicities (99.3% *vs*. 93.2%; *P* = 0.042) compared with those who received low intensity therapies. The occurrences of other toxicities were comparable between the groups (**Figure 1B**).

The CR rate was significantly higher in patients who received standard chemotherapies compared with those who received low intensity therapies, particularly in those older than 75 years (**Figure 1C**).

### OS

**Figure 2:**
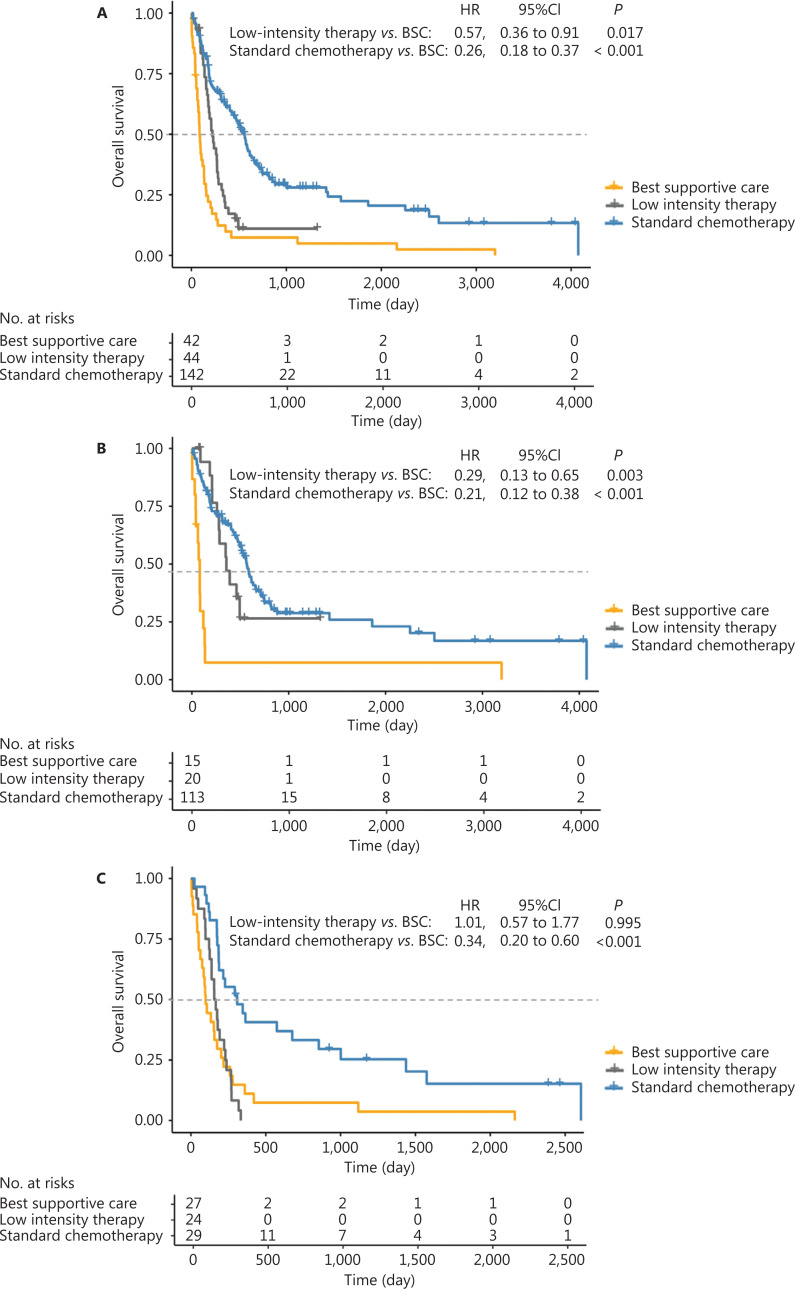
Overall survival after different treatment patterns of (A) total patients, (B) 60–75 years, and (C) beyond 75 years.

The median OS of the total population was 293 days [95% confidence interval (CI): 213–373 days]. The median OS was 561 days (95% CI: 471–650 days) and 222 days (95% CI: 165–279 days) for the standard chemotherapy and low intensity therapy groups, respectively, which were both significantly longer than that of the BSC group (86 days, 95% CI: 65–107 days; **Figure 2A**). In patients who were 60–70 years old, the OS of the standard chemotherapy and low intensity therapy groups was better than that of the BSC group (**Figure 2B**). In those older than 75 years, only the standard chemotherapy group showed better OS than the BSC group (**Figure 2C**).

**Figure 3:**
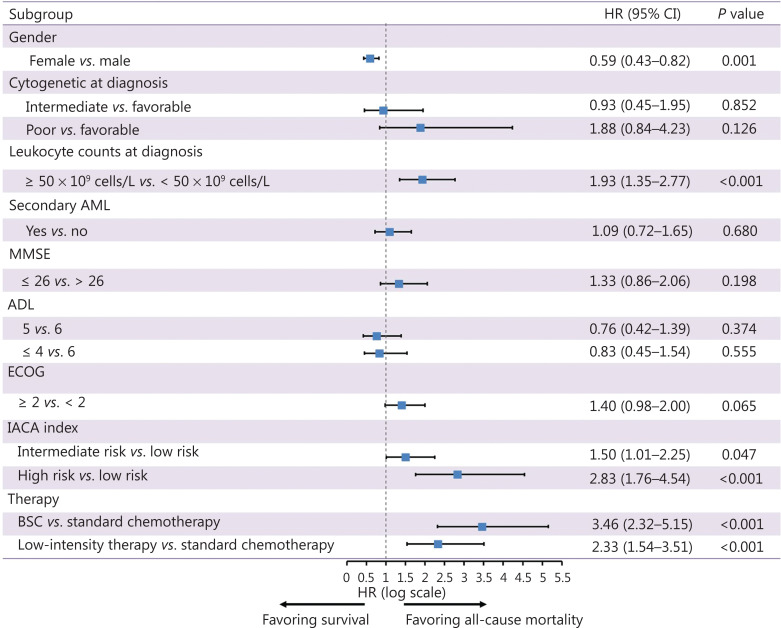
Hazard ratios and 95% confidence intervals for overall survival using multivariate analysis.

Using multivariate analysis, receiving standard chemotherapy improved the OS compared with those receiving BSC. Besides the treatment patterns, IACA intermediate and high risk status, male sex, and hyperleukocytosis at diagnosis were independently associated with a poorer OS (**Figure 3**).

### Prognostic scoring systems for elderly AML patients

We proposed a prognostic scoring system based on the results of multivariate analysis. A weighted score of 1 was assigned to male sex, IACA intermediate risk status, and hyperleukocytosis at diagnosis, and a weighted score of 2 was assigned to IACA high risk status. The overall score ranged from 0–4, with higher scores indicating greater risk. The survival rates were comparable between groups with scores of 0–1 and scores of 3–4 (data not shown). Based on these findings, we created a 3 category system: low risk, score ≤ 1; intermediate risk, score 2; and high risk, score ≥ 3. Among the 186 patients who received therapies, 79, 77, and 30 patients were categorized into low, intermediate, and high risk groups, respectively.

### The efficacy of the prognostic scoring system for elderly AML patients

**Figure 4:**
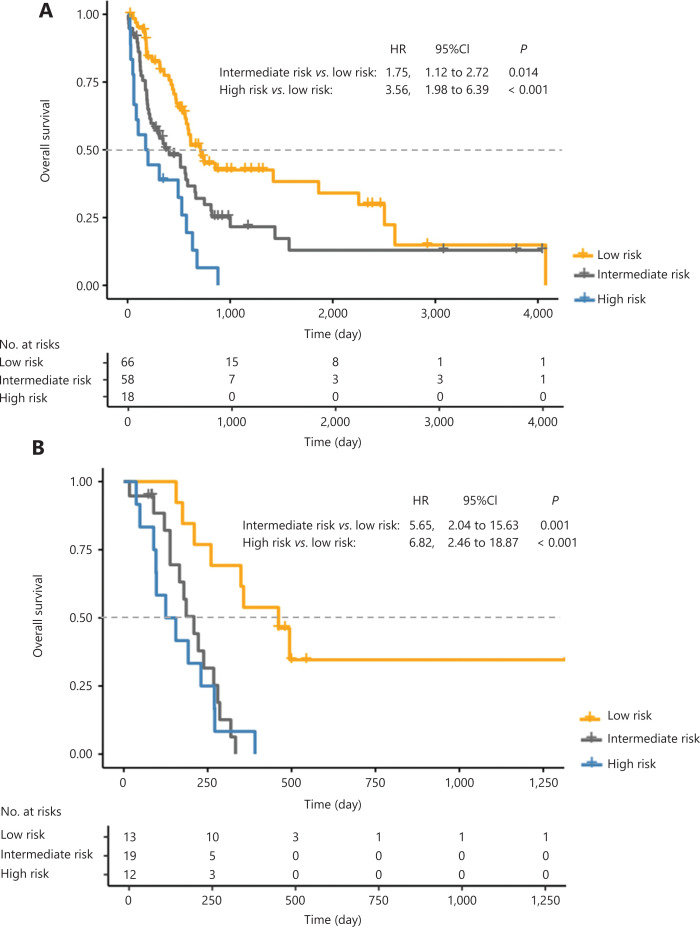
Prognostic scoring system predicted the overall survival of elderly acute myeloid leukemia patients, including (A) the standard chemotherapy group and (B) the low intensity therapy group.

Among the patients receiving standard chemotherapies, the median OS was 711 days (95% CI: 449–973 days) and 406 days (95% CI: 172–640 days) for the low risk and intermediate risk groups, respectively (*P =* 0.012), which were both significantly longer than that in the high risk group (176 days; 95% CI: 0–373 days; **Figure 4A**). Among the patients receiving low intensity therapies, the patients in the low risk group also showed the longest OS, and the median OS was comparable between the intermediate risk and high risk groups (**Figure 4B**).

**Figure 5:**
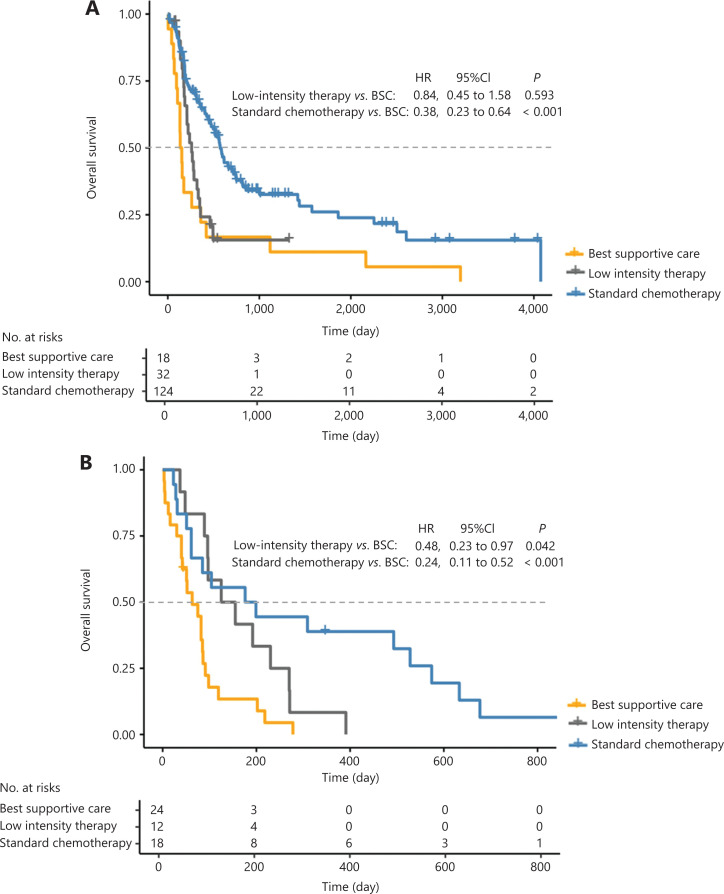
Overall survival after different treatments according to the prognostic scoring system for (A) the low and intermediate risk group, and (B) the high risk group.

Among patients in the low and intermediate risk groups, the CR rate of the standard chemotherapy group was significantly higher than that of the low intensity therapy group (67.7% *vs*. 37.5%; *P* = 0.002). The median OS of the standard chemotherapy group (573 days; 95% CI: 485–661 days) was significantly longer than that of the low intensity therapy group (260 days; 95% CI: 179–341 days; *P* < 0.001). However, the median OS was comparable between the low intensity therapy and BSC groups (136 days; 95% CI: 94–178 days; **Figure 5A**). Thus, these patients could benefit from standard chemotherapies.

Among the patients in the high risk group, the CR rate was comparable between standard chemotherapy and low intensity therapy groups (44.4% *vs*. 25.0%; *P* = 0.442). The median OS was comparable between the standard chemotherapy (176 days; 95% CI: 0–373 days) and low intensity therapy group (125 days; 95% CI: 27–223 days; *P* = 0.109), which were both significantly better than that of the BSC group (63 days: 95% CI: 27–99 days; **Figure 5B**). Hence, these patients could benefit from either standard chemotherapy or low intensity therapy, although the OS was still unsatisfactory.

## Discussion

In the present study, we found that standard chemotherapy and low intensity therapy improved survival compared with those receiving only BSC, and even in some very old patients (> 75 years). To the best of our knowledge, this was the largest multicenter study identifying the up-to-date undefined role of standard chemotherapy, low intensity therapy, and BSC in elderly Chinese AML patients. In addition, we proposed a comprehensive prognostic scoring system that could predict the OS and aid in therapeutic decisions in elderly AML patients. We could also identify a subset of elderly AML patients who would benefit more from standard chemotherapy.

In the present study, elderly AML patients who received BSC showed very poor clinical outcomes. Approximately 30% of these patients died within 8 weeks, and the median OS was only 86 days. Similarly, previous studies reported that the median OS of patients receiving BSC was only 1.5 months, 1.2 months, and 2.4 months in the United States^6^, India^31^, and Singapore^32^, respectively. Because this therapeutic technique has been significantly improved, elderly AML patients should avoid receiving BSC alone in China.

Miura et al.^33^ proposed the ACA index, including very old age (> 75 years), hypoalbuminemia, and medical comorbidities, which could predict the OS of DLBCL patients. Based on this index, Liu et al.^25^ proposed a new prognostic model, including IADL scales, and ACA index (IACA index), which could reflect the tolerance of intensive chemotherapies and predict the clinical outcomes of elderly DLBCL patients more effectively. In addition, Zhang et al.^19^ observed that the IACA index could also predict the clinical outcomes of elderly AML patients in a pilot study. In this multicenter study, the IACA index was one of the independent prognostic factors in the multivariate analysis. Advanced age at diagnosis is an important factor that could influence the survival of elderly AML patients^3,34^. Moreover, the IACA index was used to evaluate the functional status (IADL scale), nutritional status (serum albumin), and comorbidities (CCI score), which were all the key components of GA. Thus, the IACA index could consider both chronological and biological age.

Our prognostic scoring system included other variables. Hyperleukocytosis at diagnosis was associated with a higher risk of early death, which also led to a higher probability of relapse and death in the long run^30,35,36^. Thus, AML patients with hyperleukocytosis had a particularly poor prognosis. In addition, gender of patients was also associated with OS. Based on a dataset of 4,865 patients diagnosed with AML, Hossain et al.^37^ observed that females showed a significant survival advantage over their male counterparts.

Thus, our prognostic scoring system including the leukemia burden, demographic characteristics, and GA, could comprehensively reflect the status of elderly AML patients. In particular, most of the contents were objective, which could help complete the evaluations more accurately. In addition, this prognostic scoring system was easy to use and could be completed in a short time. Owing to the shortage of physicians and long waiting time for visitors, treatment has become one of the critical challenges in China^38^, so this concise scoring system could be easily popularized, particularly for doctors from general hospitals or community clinics.

For the low and intermediate risk patients in our prognostic scoring system, they might not have hyperleukocytosis. Even if they had, the tolerance of intensive therapies was relatively good (IACA low and intermediate risk). Thus, these patients could benefit more from standard chemotherapy, which could improve both the CR rate and long-term survival. It is suggested that advanced age alone is not an absolute contraindication to standard chemotherapies. For high risk patients, most may have both hyperleukocytosis and poor tolerance to intensive therapies, and therapeutic decisions should balance leukemic clearance and therapeutic toxicities. We observed that the disparity of OS between low intensity therapy and standard chemotherapy was not significant in high risk patients. Considering the toxicities of standard chemotherapy, low intensity therapy might be reasonable for these patients. However, the OS of standard chemotherapy and low intensity therapy was still unsatisfactory.

Emerging new drugs provide alternative therapeutic options for elderly AML patients^39^. HMAs have been identified in elderly AML patients who were not fit to receive standard chemotherapy, with a median OS of 5.5–9 months and a 1 year survival rate of 28%^6,7,40–42^. FLT3 inhibitors^43,44^, Bcl-2 inhibitors^45^, and IDH2 inhibitors^46^ could also be used as monotherapies in elderly AML patients. Recently, excellent outcomes of several combination therapies have been reported, particularly venetoclax combined with HMAs^47^. The median duration of CR was 11.3 months and the median OS was 17.5 months^48^. However, venetoclax has not yet been commercially available in China. Therefore, only 1 patient received venetoclax as monotherapy in the present study. In addition, all trans-retinoic acid in combination with decitabine^49,50^ and azacitidine in combination with nivolumab^51,52^ also have appeared to be safe and effective therapies for elderly AML patients. However, none of the patients received these combination therapies in the present study. New drugs could play a bigger and better role in the treatment of elderly AML patients, particularly for high risk patients according to our prognostic scoring system, because of their poor tolerance to standard chemotherapies. Knowing that the sample of patients receiving low intensity therapies was relatively small and many new drugs were not included, it would be premature to make conclusions regarding the superiority of standard chemotherapies over low intensity therapies in the present study. In the future, prospective randomized controlled trials could further compare the efficacy and safety of standard chemotherapies and new drugs in elderly AML patients.

This study had several limitations. First, it was a retrospective study, which might have influenced the accuracy of our findings. Second, patients received different therapeutic regimens in the present study, and prospective studies using unified therapeutic regimens could help to further identify the efficacy of our prognostic scoring system.

## Conclusions

We found that the clinical outcomes of patients receiving BSC were poorer than standard chemotherapy. Standard chemotherapy could improve survival, and advanced age alone was not an absolute contraindication. Our prognostic scoring system, which is a concise but comprehensive model, could predict survival and help select appropriate therapies for elderly AML patients. Standard chemotherapy is important for elderly AML patients, particularly for those categorized into low and intermediate risk groups. Considering that only a limited number of new drugs are commercially available in China, the present study has important practical significance.
