# Supplementary material for: Treatment patterns and a prognostic scoring system for elderly acute myeloid leukemia patients: a retrospective multicenter cohort study in China
Source: Cancer Biol Med. 2021 Aug 27;19(6):871–83. doi: 10.20892/j.issn.2095-3941.2020.0474 (PMC9257316; doi:10.20892/j.issn.2095-3941.2020.0474)
Supplement: Supplementary file 1 [file cbm-19-871-s001.pdf]

Supplementary materials

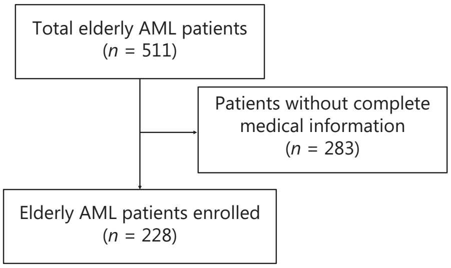

Figure S1 Diagram of patients enrolled. Patients without complete medical information were excluded.

Table S1 IACA index

Prognostic variables	IACA value		
	0	1	2
IADL scale	8	6 to 7	≤ 5
Age (years)	≤ 75	> 75	
CCI score	< 3	≥ 3	
Serum albumin (g/dL)	≥ 3.4	< 3.4	

Low risk, score 0; intermediate risk, scores 1–2; high risk, scores ≥ 3. CCI, Charlson Comorbidity Index; IACA index, IADL scales, Age, Comorbidities, and Albumin index; IADL, instrumental activities of daily living.

Table S2 Characteristics for comorbidities at diagnosis

Comorbidities	*n* (%)
Myocardial	
Angina	36 (15.8)
Myocardial infarction	8 (3.5)
Valvular	2 (0.9)
Arrhythmia	25 (11.0)
Congestive heart failure	2 (0.9)
Vascular	
Peripheral vascular disease	14 (6.1)
Hypertension	101 (44.3)
Cerebrovascular disease	30 (13.2)
Pulmonary	
Asthma	2 (0.9)
Idiopathic pulmonary fibrosis	4 (1.8)
Chronic obstructive pulmonary disease	5 (2.2)
Endocrine	
Diabetes	50 (21.9)
Diabetes with end-organ	2 (0.9)
Thyroid disease	4 (1.8)
Renal	
Chronic kidney disease	
Stage 1-2	5 (2.2)
Stage ≥ 3	20 (8.8)
Renal calculus	4 (1.8)
Hepatobiliary disease	
Chronic hepatitis B	5 (2.2)
Fatty liver	3 (1.3)
Cholelithiasis	9 (3.9)
Gastrointestinal	
Peptic ulcer	7 (3.1)
Cancer	
Solid tumor	22 (9.6)
Solid tumor with metastasis	1 (0.4)
Hematology malignancy	
Lymphoma	2 (0.9)
Leukemia	1 (0.4)
Myeloma	2 (0.9)
Autoimmune disease	
Rheumatoid arthritis	2 (0.9)
Others	3 (1.3)
Mental illness	
Anxiety	2 (0.9)
Depression	2 (0.9)
